# AdipoRon Promotes the Osseointegration of Dental Implants in Mice With Type 2 Diabetes Mellitus

**DOI:** 10.3389/fphys.2021.697738

**Published:** 2021-09-09

**Authors:** BoRui Huang, Wei Bi, Yang Sun, Ruixue Li, Xingwen Wu, Youcheng Yu

**Affiliations:** Department of Dentistry, Zhongshan Hospital, Fudan University, Shanghai, China

**Keywords:** AdipoRon, osseointegration, dental implants, type 2 diabetes mellitus, micro-CT

## Abstract

AdipoRon is an oral active synthetic small molecule with biological functions similar to adiponectin (APN). It is an APN receptor agonist that can improve insulin resistance and glucose intolerance. However, the role of AdipoRon in bone metabolism and related molecular mechanisms remains to be investigated. To explore the effect of AdipoRon on bone absorption and bone integration of type 2 diabetes mellitus (T2DM) mice with implants, we established surgery-induced model of osseointegration of dental implantation in T2DM mice of C57BL/6 db/db and normal mice homologous to diabetic mice. Micro-CT was used to analyze the femurs with the implant in the mice to detect the bone mass, H&E, and tartrate-resistant acid phosphatase (TRAP), and Safranin O-fast green staining was performed to analyze the bone formation and bone resorption. Bone integration-related markers as Rankl, bone morphogenetic protein 2 (BMP2), osteoprotegerin (OPG), osteopontin (OPN), and runt-related transcription factor 2 (Runx2) were also measured using immunohistochemistry. Our results indicated that diabetic mice showed a lower bone mass and decreased the osteoblast differentiation. AdipoRon attenuated diabetes-impaired bone volume (BV)/total volume (TV), trabecular thickness (Tb.Th), trabecular number (Tb.N), trabecular separation (Tb.Sp), and bone integration-related markers variation and promoted bone hyperplasia as well as repressed the osteoclast formation, especially in diabetic mice. AdipoRon may improve the osseointegration of dental implants in mice with T2DM by promoting osteogenesis and inhibiting bone resorption, and AdipoRon may serve as a promising oral strategy to improve the osseointegration ability of patients with diabetes.

## Introduction

Dental implants are applied for the rehabilitation of partially edentulous patients. Poor oral hygiene along with smoking and a history of periodontitis correlated with peri-implant disease strongly. Moreover, periodontitis with diabetes has been considered as the sixth major complication of diabetes, and it will lead to loosening and falling out of the teeth of patients and seriously affect their physiological functions such as chewing, swallowing, nutritional intake, speech, and facial expression (Chee et al., 2013). For tooth loss caused by periodontitis, implant treatment is the most comfortable and convenient way to repair it. As previously reported, the implant-supported restorations survival rates are related to complicated factors (Tatarakis et al., 2014). The implants may be affected by many biological complications after the initial integration phase, among which the progressive implant bone loss is generally caused by peri-implant diseases, especially by peri-implantitis with increasing high prevalence as implant therapy is implemented widespread (Lindhe et al., 2008). Moreover, there are studies concerning that type 2 diabetes mellitus (T2DM) patients with dental implants showed very similar psychosocial profiles, clinical as well as microbiological, and salivary biomarkers to those of non-diabetic individuals (Tatarakis et al., 2014), and previous researchers have confirmed that diabetic patients with poor blood glucose control have a higher failure rate of implant repair (Mellado-Valero et al., 2007).

Type 2 diabetes mellitus is a metabolic disorder characterized by hyperglycemia and lipid metabolism changes along with many complications. At present, a lot of patients with T2DM arose with an aging population and the increasing prevalence of obesity (Zheng et al., 2018; Ortega et al., 2020). Numerous previous studies indicate that macrophages in the adipose tissue interstitium of T2DM might produce a large number of pro-inflammatory factors (Chawla et al., 2011; Esser et al., 2014), which may affect bone integration. Moreover, hyperglycemia may change the biological function of bone cells to affect bone formation, bone mineralization, and bone reconstruction (Javed and Romanos, 2009), increasing the osteoclast activity and promoting the bone resorption (Catalfamo et al., 2013) to weaken the osseointegration ability of implants in patients with T2DM (Mellado-Valero et al., 2007). Therefore, poor blood glucose control is a high-risk factor for oral implant treatment, and the development of an intervention strategy that can not only effectively control blood sugar but also improve the function of damaged bone cells, so as to improve the osseointegration ability of implants, is particularly important for improving the probability of implantation success in patients with diabetes.

AdipoRon is an oral synthetic small molecular compound that was reported to bind to adiponectin (APN) receptors, specifically, AdipoR1 and AdipoR2. APN was considered to improve insulin resistance and antagonize diabetes, and it acted as an anti-inflammatory factor. APN is reported to play an important role in bone and associated with whole-body energy homeostasis, and it acts as a regulator of bone metabolism to negatively correlate with bone mineral density (Naot et al., 2017). AdipoRon can act as a potent APN receptor agonist and a potential alternative to replace APN. It was reported as an anticancer molecule to affect cell cycle progression and promote cell death in osteosarcoma cells (Sapio et al., 2020). In another report, AdipoRon can promote apoptosis while suppressing cell proliferation in myeloma cell lines (Wang S.J. et al., 2020). According to the study by Wang Z. et al. (2020), AdipoRon promoted new bone formation in diabetes mediated by impaired endochondral ossification (ECO)-induced delayed bone repair. In our previous study, we also found that AdipoRon could activate the endogenous receptors of APN to affect bone anabolism in mice with T2DM-related periodontitis and may serve as an effective multipronged approach to target periodontitis correlated with T2DM (Wu Y.C. et al., 2019). The role of AdipoRon in the osseointegration of T2DM individuals with dental implants has not been reported so far.

In the present study, the effect of AdipoRon on bone absorption and bone integration in diabetic mice along with normal control animals implanted with dental implants in the femur was explored, which may provide a new idea for enhancing the oral osseointegration ability of patients with diabetes.

## Materials and Methods

### Model of Osseointegration of Dental Implantation Induced by Surgery in Mice and Group

All the animal experiments were conducted and approved by the Animal Ethics Committees of Fudan University. Surgery-induced osseointegration of dental implantation model using male C57BL/6 db/db mice (diabetes), 6–8 weeks old, 28 ± 2 g, with fasting blood glucose (FBG) (16 h) higher than 7.0 mmol/L (FBG: 7.7 ± 1.8), and wild-type born within the same brood as control (FBG: 4.9 ± 1.3). The mice were intraperitoneally injected ketamine (100 mg/kg)/xylazine (10 mg/kg) for anesthesia and then surgical procedures were performed under sterile conditions. According to the previous study (Liu et al., 2017) with some modifications, the mice were fixed on the operating table in the supine position with skin on the lower one-third of the femur and the upper one-third of the tibia on both sides of the mouse being prepared, and the operation area was disinfected. Then a longitudinal incision of about 10 mm was made from the distal end of the femur on the medial side of the knee joint, and the incision reached the bone surface (BS). The skin and subcutaneous tissue were bluntly separated to expose the articular surface of the epiphysis, whereas the femoral periosteum was fully protected. The joint of mice was bent and the intercondylar fossa was selected as the implantation site. The implant socket with a parallel direction to the long axis of the femur and toward the proximal medullary cavity was prepared using a 25G needle under the cold condition of physiological saline. Later, the implant titanium rod with 0.5 mm in diameter and 10 mm in length from Straumann A.G. Co. (Basel, Switzerland) was tapped until its end entered slightly below the joint surface 1 mm with initial stability as well as without loosening. The peeled structure around the knee joint was reset, layered, and tightly sutured, and the window was closed. Mice were given intramuscular injections of antibiotics for three consecutive days after the operation to prevent infection.

Three days after implantation, 14 diabetic mice were divided into 2 groups with 7 mice in each group, i.e., diabetes control (D-control) with the equal volume of vehicle and diabetic mice were given 75 mg/kg/day AdipoRon (Life Sciences, United States) by intravenous administration for 2 weeks (D-AdipoRon) (Wu X. et al., 2019), and 14 wild-type mice (normal mice) were divided into 2 groups with 7 mice in each group, i.e., wild control (W-control) with the equal volume of vehicle and wild-type mice were intragastrically administrated with 75 mg/kg/day AdipoRon for 2 weeks (W-AdipoRon).

After the treatment of 2 weeks, the femurs of mice were fixed in 4% paraformaldehyde and then maintained in 75% ethanol according to the previous study (Du et al., 2020). Bone mass of femurs with the plant in the mice was analyzed using micro-CT (SkyScan 1172, Bruker) and then the femurs were harvested, fixed, decalcified, paraffin-embedded, and sectioned for pathological examination. The sections in the region surrounding the explant were selected for analysis.

### Micro-CT

Micro-CT (SkyScan 1172, Bruker) was used to analyze the femurs with the plant in the mice among all the groups to detect the volume and structure of trabecular bone. The micro-CT parameters were as follows: diameter, 21.5 mm; pixel size, 10 μm per pixel; high resolution; 212 tier; temporal integration, 250 ms; and continuous beam rotation. Set thresholds to distinguish between different organizations are as follows: gray scale values between 215 and 700 indicate trabeculae, values between 700 and 1,000 is the implant, and values less than 215 indicate non-mineralized tissue. After scanning, CT-analyzer (version: 1.15.4.0)^1^ was used for 3D reconstruction. The 0.5 mm tubular area around the implant was selected as the area of interest, the rebuild threshold to 1,000 was set. The CT-analyzer was used to perform bone histometric analysis, including trabecular thickness (Tb.Th), trabecular number (Tb.N), trabecular separation (Tb.Sp), BS/bone volume (BV), and BV/total volume (BV/TV).

### H&E Staining

After micro-CT scan, femurs were decalcified in ethylenediaminetetraacetic acid (EDTA) (10%) for 3 weeks with implant, the implant was removed and embedded in paraffin, and 5 μm sections were obtained. H&E staining was performed according to the previous study (Yang et al., 2011). In brief, the sections of femurs were dewaxed and hydrated, and then hematoxylin staining was performed. Finally, images were obtained under a microscope (Eclipse Ci-L; Nikon, Tokyo, Japan).

### Tartrate-Resistant Acid Phosphatase Staining

Based on the previous study (Du et al., 2020), sections of femur samples from mice were stained using a commercial kit of tartrate-resistant acid phosphatase (TRAP) (Sigma) according to the instruction of the manufacturer. Preosteoclasts and osteoclasts were identified by TRAP-positive cells and then counted and photographs were taken using a microscope (Nikon, Eclipse Ci-L, Japan). TRAP-positive cells in the peri-implant were detected by the presence of dark-purple staining granules in the cytoplasm. Image-Pro Plus 6.0 was used for TRAP staining analysis, and five fields of vision were randomly selected in each section. The percentage of TRAP-positive cells is calculated as the TRAP-positive stained area/total area × 100%, which is used to indicate osteoclasts.

### Safranin O-Fast Green Staining

Safranin O-fast green staining was performed to observe osteogenesis as reported in a previous study (Pritzker et al., 2006). In brief, the sections of femur samples from mice were stained in fast green dye solution for 5–10 min and then washed followed by staining in safflower dye solution for 15–30 s. After being sealed, the sections were observed under a microscope along with images acquisition and analysis. The cartilage is red or salmon-red with a green background. CaseViewer2.2 Scanning Browser software was used to select the target area of the tissue for imaging. The Safranin O-positive cartilage was analyzed by ImagePro Plus 6.0 (Media Cybernetics, Bethesda, MD, United States).

### Immunohistochemistry

Immunohistochemistry was used to detect the expressions of Rankl, bone morphogenetic protein 2 (BMP2), osteoprotegerin (OPG), osteopontin (OPN), and runt-related transcription factor 2 (Runx2) in femoral bone with dental implants in mice. The sections of femur samples from mice were incubated in hyaluronidase and skimmed milk for blocking. And then the sections were incubated with primary rabbit polyclonal antibodies to Rankl (Abcam, CN. ab216484, 1:100), BMP2 (ZEN BIO, CN. 500231, 1:100), OPG (Abcam, CN. ab183910,1:100), OPN (SANTA CRUZ, CN. sc-21742, 1:100), or Runx2 (Abcam, CN. ab192256, 1:100) at 4°C overnight. After washing in phosphate-buffered saline, sections were incubated with the corresponding horseradish peroxidase (HRP) conjugated secondary antibody (1:3,000) at room temperature for 30 min followed by visualization using diaminobenzidine (DAB) and counterstaining with hematoxylin. Images were obtained using an Eclipse Ci-L microscope. Image-Pro Plus 6.0 software was used to analyze images. All proteins were categorized based on a histochemical score (H-score), and positive comprehensive scores were obtained from five visual fields in each section according to the same criteria. A numerical value from a weighted summation of percentage staining accounts for both the staining intensity and the percentage of cells at that intensity.

### Data Statistics

GraphPad Prism (8.0, United States) was used to analyze the data that were obtained from at least three independent samples and presented as the mean ± standard deviation (SD). Two-way repeated-measures analysis of variance (ANOVA) was used to analyze the data from three or more groups and an unpaired *t*-test was used to analyze the data from two groups. When *p*-value is ≤0.05, differences were considered statistically.

## Results

### AdipoRon Promotes Bone Formation Around Dental Implants in Mice

**FIGURE 1:**
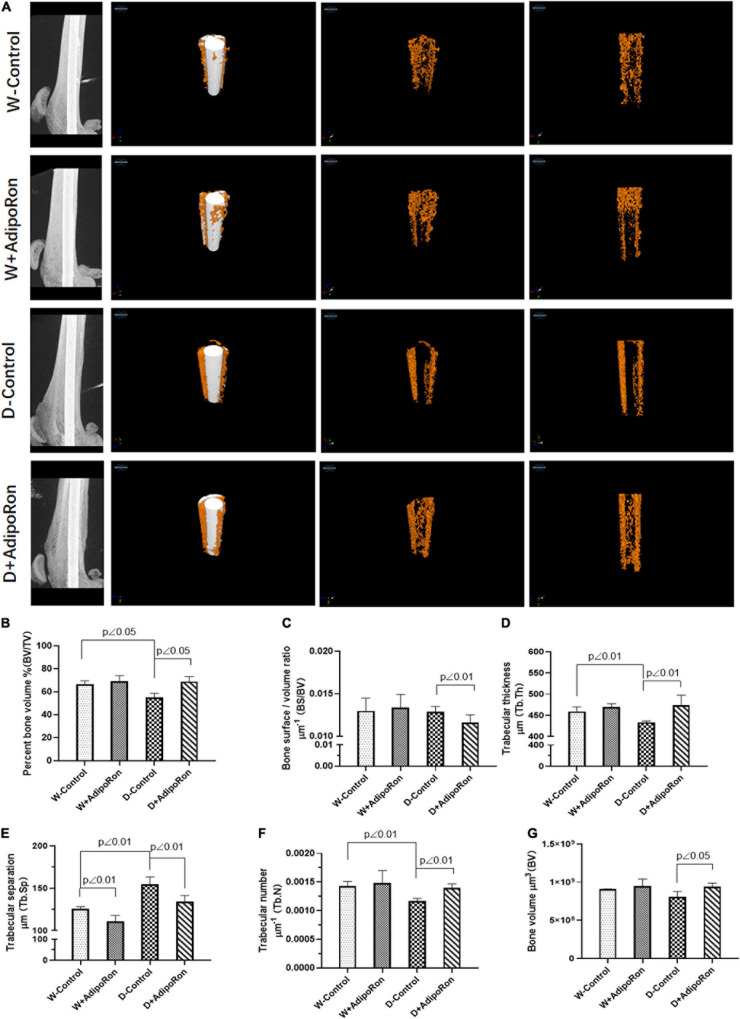
Evaluation of femoral bone structure with dental implants in mice. Femoral bone structure with dental implants in mice was assessed using micro-CT (*n* = 7). Normal mice (Wild-control, W-control), normal mice treated with AdipoRon (Wild + AdipoRon, W + AdipoRon), diabetic mice (Diabetes-control, D-control), and diabetic mice treated with (Diabetes + AdipoRon, D + AdipoRon) were involved. **(A)** 3D reconstruction images of femoral bone structure with dental implants in mice are shown. Values of bone volume/tissue volume (BV/TV) **(B)**, bone surface/bone volume (BS/BV) **(C)**, trabecular thickness (Tb.Th) **(D)**, trabecular separation (Tb.Sp) **(E)**, trabecular number (Tb.N) **(F)**, and bone volume (BV) **(G)** were analyzed.

Bone morphology and formation of mice were observed using micro-CT. Results of micro-CT detection are shown in Figure 1. Diabetic mice (Diabetes-control, D-control) showed lower bone mass around dental implants compared with the normal mice (Wild-control, W-control). The 3D reconstruction images from micro-CT are shown in Figure 1A, BV/TV in Figure 1B, and Tb.Th in Figure 1D. Also, Tb.N (Figure 1F) was significantly decreased, whereas Tb.Sp (Figure 1E) was increased in diabetic mice compared with normal mice. AdipoRon could attenuate the induction of diabetes in BV/TV, Tb.Th, Tb.N, and Tb.Sp. Although there was almost no difference between diabetic mice and the normal mice, AdipoRon treatment decreased BS/BV (Figure 1C) along with increased BV (Figure 1G) of diabetic mice. Diabetic mice showed lower bone mass around dental implants compared with normal mice, which may be attenuated by AdipoRon.

### AdipoRon Promotes the Osteoblast Differentiation and Inhibits Osteoclast

**FIGURE 2:**
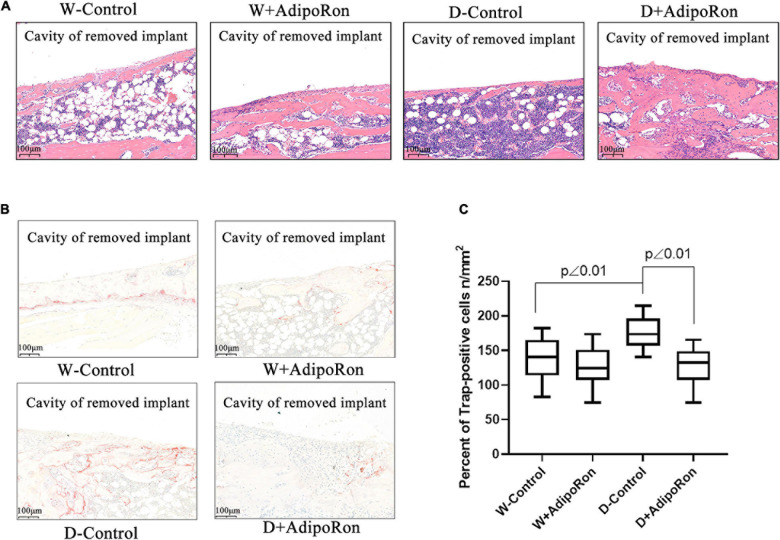
Pathological examinations of femoral bone with dental implants in mice. Femoral bone pathology was observed. Normal mice (Wild-control, W-control), normal mice treated with AdipoRon (Wild + AdipoRon, W + AdipoRon), diabetic mice (Diabetes-control, D-control), and diabetic mice treated with (Diabetes + AdipoRon, D + AdipoRon) were involved. *n* = 7. **(A)** H&E staining of femoral bone with dental implants in mice was shown. One section in the region surrounding the explant of each sample was selected for analysis and five fields of vision were randomly selected in each section. The green arrow indicates bone hyperplasia mediated by the osteoblast differentiation. **(B)** TRAP staining of femoral bone with dental implants in mice is shown. The blue arrow indicates the osteoclast formation. **(C)** TRAP-positive cells of osteoclast were analyzed.

Pathological exploration of femoral bone after dental implantation and AdipoRon treatment in mice was performed. As shown in Figure 2A, bone hyperplasia mediated by the osteoblast differentiation was significantly decreased in diabetic mice (Diabetes-control, D-control) compared with normal mice (Wild-control, W-control). AdipoRon promoted bone hyperplasia both in normal and diabetic mice, especially in the latter. The osteoclast formation indicated by TRAP-positive staining was significantly increased in D-control compared with W-control, whereas the osteoclast formation decreased significantly (*p* < 0.05) in both the AdipoRon-treated diabetic mice (Diabetes + AdipoRon, D + AdipoRon) and the normal mice (Wild + AdipoRon, W + AdipoRon) (Figures 2B,C).

### AdipoRon Represses the Osteoclast Formation of Safranin O-Fast Green Staining

**FIGURE 3:**
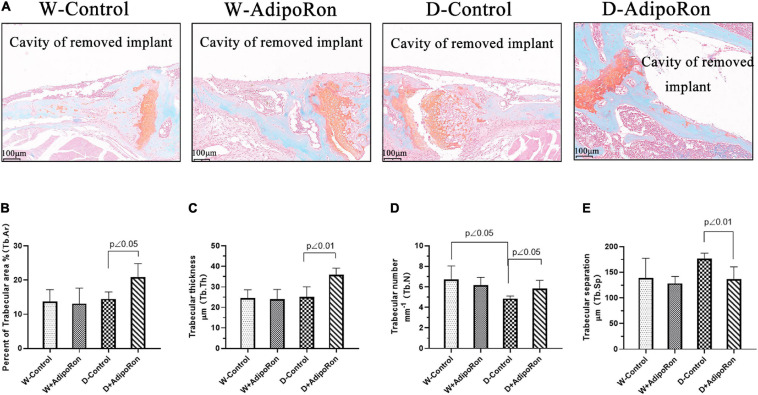
The osteoclast formation was measured using Safranin O-fast green staining. Normal mice (Wild-control, W-control), normal mice treated with AdipoRon (Wild + AdipoRon, W + AdipoRon), diabetic mice (Diabetes-control, D-control), and diabetic mice treated with (Diabetes + AdipoRon, D + AdipoRon) were involved (*n* = 7). **(A)** Femoral bone with dental implants in mice was stained using Safranin O-fast green. One section in the region surrounding the explant of each sample was selected for analysis and five fields of vision were randomly selected in each section. Percentage of trabecular area (Tb.Ar) **(B)**, trabecular thickness (Tb.Th) **(C)**, trabecular number (Tb.N) **(D)**, and trabecular separation (Tb.Sp) **(E)** were analyzed.

The osteoclast formation in femoral bone with dental implants in mice was further investigated using Safranin O-fast green staining. Results were shown in Figure 3 that Tb.N was decreased in diabetic mice (Diabetes-control, D-control) compared with normal mice (Wild-control, W-control) (Figures 3A,D) although there was no difference in the percentage of the trabecular area (Tb.Ar) (Figure 3B), Tb.Th (Figure 3C), and Tb.Sp (Figure 3E) among all the mice involved in W-control and D-control which was slightly different from the results from micro-CT. AdipoRon increased the values of Tb.Ar, Tb.Th, and Tb.N, but decreased Tb.Sp in diabetic mice. Safranin O-fast green staining results confirmed that AdipoRon may suppress the osteoclast formation.

### AdipoRon Suppresses Proteins of Rankl as Well as Promotes the Expressions of BMP2, OPG, OPN, and Runx2 in Diabetic Mice

**FIGURE 4:**
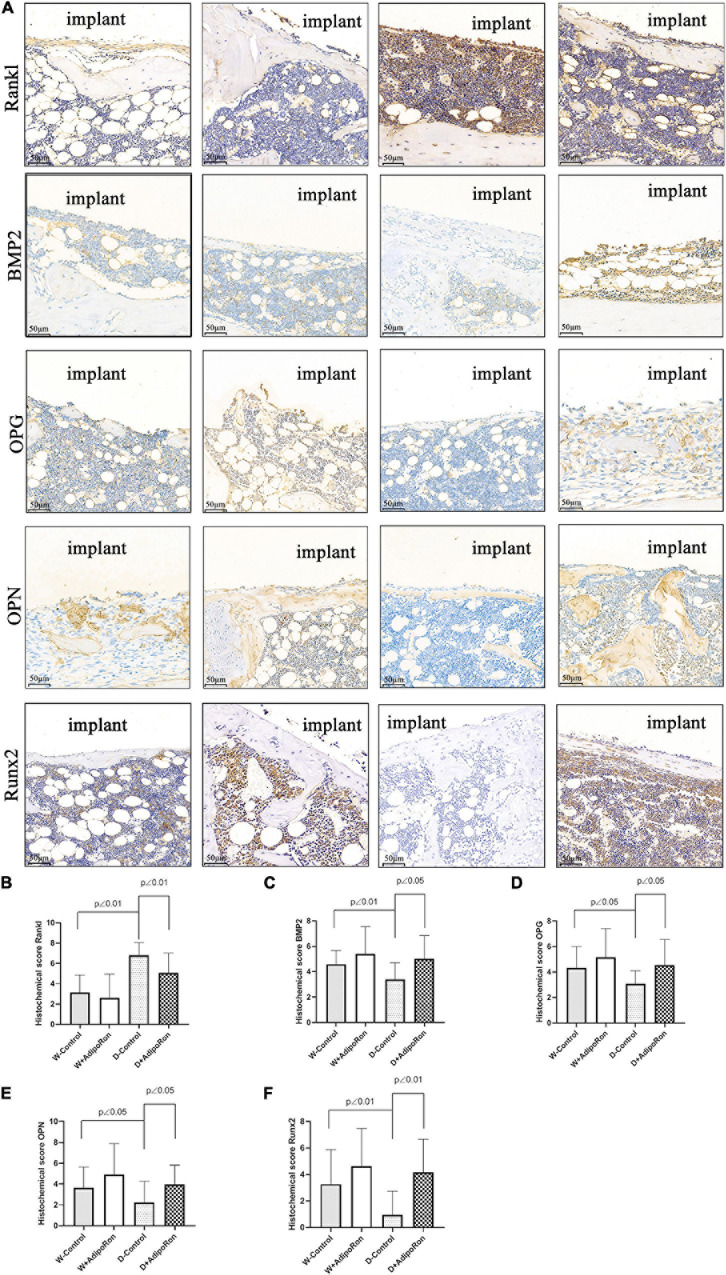
Proteins of Rankl, BMP2, OPG, OPN, and Runx2 in femoral bone with dental implants in mice were quantized using immunohistochemical staining. Normal mice (Wild-control, W-control), normal mice treated with AdipoRon (Wild + AdipoRon, W + AdipoRon), diabetic mice (Diabetes-control, D-control), and diabetic mice treated with (Diabetes + AdipoRon, D + AdipoRon) were involved (*n* = 7). **(A)** Femoral bone with dental implants in mice was immunohistochemically stained. One section in the region surrounding the explant of each sample was selected for analysis and five fields of vision were randomly selected in each section. Quantitation of Rankl **(B)**, BMP2 **(C)**, OPG **(D)**, OPN **(E)**, and Runx2 **(F)** are indicated using histochemical scores.

The expressions of proteins, such as Rankl, BMP2, OPG, OPN, and Runx2, in femoral bone with dental implants in mice were measured using immunohistochemical staining. As shown in Figure 4, the expression of protein Rankl (Figures 4A,B) was significantly increased and the expressions of proteins BMP2 (Figures 4A,C), OPG (Figures 4A,D), OPN (Figures 4A,E), and Runx2 (Figures 4A,F) were significantly decreased in diabetic mice (Diabetes-control, D-control) compared with normal mice (Wild-control, W-control) (*p* < 0.05). However, AdipoRon inhibited the expression of protein Rankl along with the promoted expressions of BMP2, OPG, OPN, and Runx2 significantly in diabetic mice (*p* < 0.05). Results of examination of protein expression suggested that AdipoRon suppressed the osteoclast formation and promoted the osteoblast differentiation in diabetic mice.

## Discussion

Type 2 diabetes mellitus with a lot of complications affects human life and health expenditures along with rapid economic development and urbanization (Onyango and Onyango, 2018). Periodontitis is one of the major complications of diabetes including teeth loosening and falling, which affects the physiological functions of teeth of patients seriously (Chee et al., 2013). Although implant treatment is the most important way to repair the lost tooth, hyperglycemia induced a higher failure rate of implant repair (Mellado-Valero et al., 2007). Macrophages in the adipose tissue of T2DM patients correlated with bone integration including bone formation, bone mineralization, and bone reconstruction (Javed and Romanos, 2009) as well as osteoclast activity and promote bone resorption (Catalfamo et al., 2013) and hyperglycemia may impair bone integration of T2DM patients with implants. Along with imposing restrictions on hyperglycemia, improving the function of damaged bone cells and the osseointegration ability of implants is particularly important for implantation success in patients with diabetes. We established the surgery-induced model of osseointegration of dental implantation in mice to investigate the difference in bone formation and bone resorption between diabetic mice (C57BL/6 db/db, T2DM mice) and the normal mice homologous to diabetic mice and tend to found an effective intervention strategy to implant repair loss tooth under hyperglycemia.

As one of the insulin-sensitizing fatty factors, APN is secreted by fat cells to improve insulin resistance and attenuate hyperglycemia as well as impair RANKL-stimulated RAW264.7 cells through its receptors as AdipoR1, AdipoR2 (Okada-Iwabu et al., 2013), and T-Cadherin (Denzel et al., 2010; Parker-Duffen et al., 2013) and its downstream factors of cohesive protein APPL1 (Mao et al., 2006). APN can also promote osteogenic differentiation (Chen et al., 2015; Pu et al., 2016). However, the clinical application of APN was limited as potential adverse immune reactions, deserving high-dose intravenous administration and complex protein structures are not conducive to industrial production. AdipoRon is an oral synthetic small molecular compound as it has a feasible effect on reducing insulin resistance and blood glucose tolerance in high-fat diet mice by specifically binding to APN receptors AdipoR1 and AdipoR2. AdipoRon might exert similar antidiabetic effects to APN through AMPK and PPAR-α pathway. Some studies also indicated that AdipoRon could promote diabetic fracture repair, enhance alveolar bone regeneration, and increase the survival and migration of BMSCs. According to a recent study, AdipoRon appears to have a pro-osteogenic, anti-adipogenic, and anti-osteoclastogenic effect in young mice (Liu et al., 2021).

We explored the effect of AdipoRon on bone formation and bone resorption in diabetic mice. Micro-CT and pathological examination using H&E, TRAP, and Safranin O-fast green staining were performed to evaluate bone integration. Our results indicated that diabetic mice showed lower bone mass around dental implants compared with the normal mice as BV/TV, Tb.Th, and Tb.N was significantly decreased compared with normal mice. Results of pathological exploration by H&E staining of femoral bone showed that bone hyperplasia mediated by the osteoblast differentiation was significantly decreased in diabetic mice compared with normal mice. As shown in Safranin O-fast green staining results, the osteoclast formation in femoral bone with dental implants in diabetic mice was improved as Tb.N was decreased compared with normal mice. AdipoRon attenuated diabetes-impaired BV/TV, Tb.Th, Tb.N, and Tb.Sp variation and promoted bone hyperplasia as well as repressed the osteoclast formation, especially in diabetic mice.

Rankl is positively related to osteoclast differentiation in periodontitis (Li et al., 2020). Down expression of BMP2 and RUNX2 is involved in reducing osteogenic differentiation (Kang et al., 2020). OPG is a physiological inhibitor of RANKL, both of which regulated the delicate bone balance involving the interplay of soluble mediators. As a decoy receptor, OPG could prevent RANKL from binding to RANK exerting an osteoprotective effect (Dereka et al., 2010). OPG was also reported as one of the factors in predicting the severity of gestational diabetes mellitus, and it is considered as OPG presents a high precision potential in the identification of periodontal disease destruction (Hernandez et al., 2020). OPN accompanied with RUNX2 showed an increased expression as high glucose-inhibited osteogenesis of periodontal ligament stem cells was reversed (Yan et al., 2020). Therefore, bone formation and osteogenic differentiation-related markers of the expressions of Rankl, BMP2, OPG, OPN, and Runx2 were assayed in femoral bone with dental implants in mice. The expression of protein Rankl was significantly increased as well as the expressions of BMP2, OPG, OPN, and Runx2 were significantly decreased in diabetic mice compared with normal mice (*p* < 0.05). AdipoRon suppressed the expression of protein Rankl and promoted the expressions of BMP2, OPG, OPN, and Runx2 in diabetic mice significantly (*p* < 0.05), which suggested that AdipoRon suppressed the diabetes-induced osteoclast formation and promoted the osteoblast differentiation in diabetic mice. These results were consistent with our previous study (Wu Y.C. et al., 2019). AdipoRon may improve insulin resistance by activating AdipoR1/AMPK/PGC1α signaling pathways (Kim and Park, 2019). In addition, AMPK is an important molecular target to metabolic diseases, such as diabetes, to enhance the recovery of osteoblast function and osseointegration around implants. It is necessary for us to perform further study to expound whether AdipoRon can regulate the expression of bone-related proteins through AMPK. Taking together, our results confirmed that AdipoRon may improve the osseointegration of dental implants in mice with T2DM for 2 weeks through promoting osteogenesis and inhibiting bone resorption mediated by the regulation of bone formation-related markers such as Rankl, BMP2, OPG, OPN, and Runx2. However, the enduring effect of AdipoRon and the specific molecular mechanism remains to be explored in further long-term studies. Overall, AdipoRon may serve as a promising oral strategy to improve the osseointegration ability of patients with diabetes.

## Data Availability Statement

The original contributions presented in the study are included in the article/supplementary material, further inquiries can be directed to the corresponding authors.

## Ethics Statement

The animal study was reviewed and approved by Animal Ethics Committees of the Fudan University.

## Author Contributions

BH and WB: project developing, data analysis, writing, and revision. YS: project developing and data collection. RL: data analysis and collection. XW and YY: manuscript writing, revision, and editing.

## Conflict of Interest

The authors declare that the research was conducted in the absence of any commercial or financial relationships that could be construed as a potential conflict of interest.

## Publisher’s Note

All claims expressed in this article are solely those of the authors and do not necessarily represent those of their affiliated organizations, or those of the publisher, the editors and the reviewers. Any product that may be evaluated in this article, or claim that may be made by its manufacturer, is not guaranteed or endorsed by the publisher.

## Footnotes

1
www.Skyscan.be
